# Enhancement of endothelial function and attenuation of portal vein injury using mesenchymal stem cells carrying miRNA-25-3p

**DOI:** 10.1038/s41598-024-64263-6

**Published:** 2024-07-02

**Authors:** Guole Nie, Honglong Zhang, Wei Luo, Xingwang Zhu, Danna Xie, Jun Yan, Haiping Wang, Xun Li

**Affiliations:** 1 The First School of Clinical Medicine, Lanzhou University, Lanzhou, 730000 China; 2 Department of General Surgery, The First Hospital of Lanzhou University, Lanzhou, 730000 China; 3 Key Laboratory of Biotherapy and Regenerative Medicine of Gansu Province, Lanzhou, 730000 China; 4 Cancer Prevention and Control Center of Lanzhou University Medical School, Lanzhou, 730000 China; 5 Gansu Institute of Hepatobiliary and Pancreatic Surgery, Lanzhou, 730000 China; 6 Gansu Clinical Medical Research Center of General Surgery, Lanzhou, 730000 China

**Keywords:** miRNA-25-3p, Mesenchymal stem cells, Exosomes, Human umbilical vein endothelial cells, Portal vein, Thrombosis, Liver diseases, Stem cells, Embryonic stem cells

## Abstract

The aims of this study were to determine whether human umbilical cord mesenchymal stem cells (hucMSCs) modified by miRNA-25-3p (miR-25-3p) overexpression could promote venous endothelial cell proliferation and attenuate portal endothelial cell injury. HucMSCs and human umbilical vein endothelial cells (HUVEC) were isolated and cultured from human umbilical cord and characterized. Lentiviral vectors expressing miRNA-25-3p were transfected into hucMSCs and confirmed by PCR. We verified the effect of miR-25-3p-modified hucMSCs on HUVEC by cell co-culture and cell supernatant experiments. Subsequently, exosomes of miR-25-3p-modified hucMSCs were isolated from cell culture supernatants and characterized by WB, NTA and TEM. We verified the effects of miR-25-3p-modified exosomes derived from hucMSCs on HUVEC proliferation, migration, and angiogenesis by in vitro cellular function experiments. Meanwhile, we further examined the downstream target genes and signaling pathways potentially affected by miR-25-3p-modified hucMSC-derived exosomes in HUVEC. Finally, we established a rat portal vein venous thrombosis model by injecting CM-DiR-labeled hucMSCs intravenously into rats and examining the homing of cells in the portal vein by fluorescence microscopy. Histological and immunohistochemical experiments were used to examine the effects of miRNA-25-3p-modified hucMSCs on the proliferation and damage of portal vein endothelial cells. Primary hucMSCs and HUVECs were successfully isolated, cultured and characterized. Primary hucMSCs were modified with a lentiviral vector carrying miR-25-3p at MOI 80. Co-culture and cell supernatant intervention experiments showed that overexpression of miRNA-25-3p in hucMSCs enhanced HUVEC proliferation, migration and tube formation in vitro. We successfully isolated and characterized exosomes of miR-25-3p-modified hucMSCs, and exosome intervention experiments demonstrated that miR-25-3p-modified exosomes derived from hucMSCs similarly enhanced the proliferation, migration, and angiogenesis of HUVECs. Subsequent PCR and WB analyses indicated PTEN/KLF4/AKT/ERK1/2 as potential pathways of action. Analysis in a rat portal vein thrombosis model showed that miR-25-3p-modified hucMSCs could homing to damaged portal veins. Subsequent histological and immunohistochemical examinations demonstrated that intervention with miR-25-3p overexpression-modified hucMSCs significantly reduced damage and attenuated thrombosis in rat portal veins. The above findings indicate suggest that hucMSCs based on miR-25-3p modification may be a promising therapeutic approach for use in venous thrombotic diseases.

## Introduction

Portal vein thrombosis (PVT) is a common complication in patients with liver cirrhosis, with an incidence ranging from 5 to 26%^1,2^. PVT is characterized by the development of a thrombus in the portal vein and its branches, with or without splenic or superior mesenteric vein involvement^3^. PVT may increase portal venous pressure, leading to increased risks such as bleeding from fundic esophageal vein rupture, intestinal stasis, and hepatic failure, all of which can significantly impact patient survival and prognosis^4^. Anticoagulation is an effective therapy for patients with PVT^5^. However, in patients with cirrhosis, the use of anticoagulants is carefully balanced against the risk of thrombotic recurrence and bleeding, but the possibility of PVT progression persists in some patients after anticoagulation therapy^6,7^. Consequently, identifying and treating PVT early can be challenging due to its insidious progression and lack of specific clinical manifestations. The hypercoagulable state of the blood, slow blood flow, and local vascular endothelial cell damage are key factors in PVT formation^8^. As endothelial cell dysfunction remains an important link in thrombosis^9^, repairing damaged endothelial cells and improving endothelial cell function may play a crucial role in both the onset and the development of PVT.

Stem cell therapy is a novel and important therapeutic modality in clinical medicine^10^. Human umbilical cord mesenchymal stem cells (hucMSCs) have been extensively researched for tissue repair and regenerative medicine because to its multidirectional differentiation ability and paracrine action^11^. Exosomes (Exos), small secretory membrane vesicles secreted by cells, play an important role in intercellular communication patterns by transferring molecules (such as mRNAs, miRNAs, and proteins) from one cell to another in order to regulate physiological and pathological processes^12^. The positive benefits of stem cell transplantation may be mostly due to paracrine processes^13,14^. Exos are an important component of mesenchymal stem cell paracrine secretion^15^.

MiR-25-3p belongs to the miR-106b/25 cluster. Notably, miR-106b and miR-93 share the same seed sequence, whereas miR-25 has a different seed sequence, with a wide range of projected target genes, indicating that it is involved in a variety of biological activities^16^. miR-25-3p is an endothelial cell-specific angiogenic miRNA, and cancer exo-derived miR-25-3p enhances vascular permeability and angiogenesis in human umbilical vein endothelial cells (HUVECs) in vitro^17^. Further, miR-25-3p expression is dramatically reduced in the serum of patients with acute cirrhosis decompensation, and the risk of PVT is greatly elevated in patients with acute cirrhosis decompensation^18^. Therefore, this study investigated the impact of exos of miR-25-3p-modified hucMSCs on the cellular phenotype of HUVECs in vitro and during the development of PVT in vivo.

Currently, reports regarding the use of miR-25-3p in combination with hucMSCs in the treatment of venous thrombosis remain lacking. Therefore, in this study, we used a miR-25-3p lentiviral vector to modify hucMSCs and explore their effects on the proliferation, migration, and tubule formation of HUVEC. Simultaneously, we also investigated the potential downstream target genes and mechanism of miR-25-3p modified hucMSCs. Finally, we constructed a PVT thrombus model in rats to investigate the therapeutic effects of miR-25-3p lentiviral vector-modified hucMSCs on PVT.

## Materials and methods

### Cell extraction and culture

Fresh umbilical cords were obtained from women who had just given birth and were processed quickly with informed consent from women hospitalized at the First Hospital of Lanzhou University. All experimental protocols were approved by the Ethics Committee of the First Hospital of Lanzhou University (LDYYLL-2024-01). Human umbilical cords were collected and treated as previously reported. hucMSCs and HUVECs were isolated, cultivated, cryopreserved, and passaged in the same approach as previously described. HucMSCs were cultured in DMEM (Biological Industries, Israel) containing 10% fetal bovine serum (Gibco, USA) in an incubator at 37 °C with 5% CO_2_. HUVEC were cultured in endothelial cell medium (ScIenCell, USA) containing 5% fetal bovine serum (FBS) in incubator at 37 °C with 5% CO_2_. When exosomes were collected, hucMSCs were cultured in basal medium containing 10% exosome-free FBS (Oricell, Guangzhou, China).

### Identification of hucMSCs and HUVECs

The cell surface markers (CD29, CD34, CD44, CD45, CD90, CD105, CD166, and HLA-DR) of hucMSCs were identified by flow cytometry. Multiplex lineage differentiation ability was also tested using osteogenic and lipidogenic medium (OriCell, Guangzhou China). Immunofluorescence labeling for CD31 and vWF identified HUVEC phenotypes.

### Lentiviral transfection

GenePharma (Shanghai, China) designed and synthesized the miRNA-25-3p-GFP and miRNA-25-3p-NC-GFP lentiviral vectors. The multiplicity of infection (MOI) was set to 0, 20, 50, 80, and 100, in that order. The cell were infected with lentiviral media for 24 h before being replaced with fresh medium for another 48 h of incubation. The cells were screened for 72 h in DMEM containing 10% FBS with puromycin at a dosage of 2 µg/ml. Fluorescence microscopy was used to identify the green fluorescent protein signal, and PCR was used to confirm the gene transfection effectiveness.

### Cell co-culture and cell CM intervention

Transwell chambers (0.4 m pore size, JET BIOFIL, China) were used to co-culture HUVEC and hucMSC. The upper chamber of the Transwell was filled with hUVEC suspended in ECM, while the lower chamber was filled with modified hucMSCs (miRNA-25-3p-hucMSCs or NC-hucMSCs) grown in DMEM. HUVECs in the upper compartment were harvested after 24 h of co-culture for cell function experiments.

When the hucMSCs had grown to around 60–70%, they were replaced with serum-free DMEM and cultivated for 48 h to collect the CM, which was filtered through a 0.22 pore size filter and prepared for use. Preparation of Conditioned Medium for Intervention in HUVEC (EC-CM) from Collected CM. Conditioned medium (EC-CM) for intervening HUVECs was prepared from the collected CM, and the HUVECs were collected for cell function experiments 24 h after EC-CM intervention with HUVECs.

### Endothelial proliferation assay

HUVECs proliferation was evaluated using the EDU Proliferation Kit (C0075S, Beyotime, China) according to the manufacturer's recommendations. HUVECs were equally distributed in 24-well plates for overnight incubation, and DMEM with EDU (1×) was preincubated. The cells were fixed with 4% paraformaldehyde and then permeabilized for 30 min in light-avoidance incubation with TrionX-100 and EDU reaction buffer. Finally, the nuclei of the cells were stained with Hoechst 33342 (1×) for 15 min. Three randomly selected fields of view (200×) per well were used for quantification of cell proliferation using Image J. The percentage of EDU-stained cells as compared to DAPI-stained cells was used to calculate the proliferation rate of HUVECs.

### Wound healing assay

For wound healing assay, HUVECs were harvested after inoculation into 6-well plates. When the cells grew to more than 95%, they were mechanically scratched using 200 μl pipette tips and photographed under a microscope taken as 0 h. After 24 h, defect closure images were taken using a microscope, and three randomly selected fields (100×) for each group were used to calculate the area of cell migration.

### Transwell assay

Transwell experiments were performed using a 24-well transwell cell culture chamber (8 μm wells). A serum-free mixture of ECM-HUVECs was inoculated into the upper chamber and ECM with 20% FBS was added to the lower chamber. After 24 h, the cells were fixed in 4% paraformaldehyde, stained with crystal violet and the cells on the inner side of the chambers were erased. Migrating cells on the bottom of the chambers were observed under an inverted microscope and transmembrane cells were randomly selected in three fields (100×) in each transwell chambers, imaged and counted using Image J.

### Tube formation assay

Tubule formation assay was performed to test the in vitro angiogenic capacity of HUVECs. 24-well plates were filled with 330 μl of matrix gel (082704, ABW, China) per well and placed in the incubator at 37 °C for 45 min. HUVECs were inoculated into each well at a density of 6 × 104 cells per well and incubation in the incubator at 37 °C for 4 h. Tubules were photographed and analyzed under an inverted microscope. Three randomly selected fields (100×) per well were used to quantify the number of tubes and tubule length using image J.

### Extraction and characterization of exosomes

HucMSCs-exosomes were extracted from the supernatant of cell cultures by ultracentrifugation as previously described^19^. The morphology of hucMSCs-exosomes was observed under transmission electron microscopy. Representative markers of exosomes, including CD63, CD9, and TSG101^20^, were identified by protein blotting analysis. Nanoparticle tracking analysis (NTA) was used to detect exosome diameter. To explore whether hucMSCs-exosomes could be uptaken by HUVECs, exosomes were labeled with the fluorescent dye CM-DiR and incubated with HUVECs for 4 h at 37 °C. HUVECs were fixed in 4% paraformaldehyde for 15 min and treated with DAPI for 15 min. Finally, HUVECs were observed by confocal laser microscopy.

### GW4869 inhibits exosomes release

GW4869 (UR21021, Umibio, China) is a cell-permeable, specific, non-competitive neutral sphingomyelinase 2 inhibitor that inhibits exosome release from cells. The hucMSCs were inoculated in Petri dishes and cultured until the cell fusion was 60–70%; the original cell culture medium was removed and replaced with fresh exosome-free cell culture medium containing GW4869 inhibitor (10 μM) and cultured for 24 h. The cells were co-cultured with HUVECs when the cell fusion reached 85–95%, or the supernatant was collected from the cells for the subsequent intervention experiments.

### Surgical procedures and interventions in the rat PVT model

Male SD rats, weighing about 200–250 g, were used in this experiment, purchased from the Laboratory Animal Center of Lanzhou University and approved by the Animal Care and Use Committee of the First Hospital of Lanzhou University. The rat PVT model and hucMSC intervention were performed with reference to previously described methods^21,22^. Finally, the rats were randomly divided into five groups: (1) sham-operated group, (2) PVT group, (3) PVT + hucMSCs group (hucMSCs group), (4) PVT + NC-hucMSCs group (NC-hucMSCs group), (5) PVT + miRNA-25-3p-hucMSCs group (miRNA-25-3p-hucMSCs group). All cell transplantation groups received 200 μL of PBS and 2 × 10^6^ hucMSCs or modified hucMSCs (NC-hucMSCs or miRNA-25-3p-hucMSCs) through the tail vein 2 h before PVT model formation. The PVT group received an equivalent volume of PBS. Except for the portal vein freeing, neither model creation nor hucMSC implantation were conducted in the sham-operated group.

Observation of CM-DiR-labeled hucMSCs by fluorescence microscopy (Leica, Germany) was used to check whether hucMSCs could homing to the damaged portal vein 1 day after transplantation. All experiments were performed in accordance with relevant guidelines and regulations, and approved by the Ethics Committee of the First Hospital of Lanzhou University(LDYYLL-2024-01).

### Histological and immunohistochemical examination of portal vein

Portal veins of rats were routinely collected, fixed and embedded. Hematoxylin–eosin (H–E) and Masson staining were used for morphological and histologic analysis of the portal veins. Immunohistochemical analysis was performed using the test kit according to the instructions. Portal vein tissue sections were incubated with Ki-67, TNF-α, CD68 and CD31 respectively and then re-stained with hematoxylin, and the expression of each marker was observed under the microscope.

### Western blot

Total protein was extracted from cells and exosomes by RIPA buffer, benzylsulfonyl fluoride (PMSF) and phosphatase inhibitors. Total proteins were quantified using the BCA Protein Assay Kit and separated by SDS-PAGE and transferred to PVDF membranes. The PVDF membrane was closed using 5% BSA. The PVDF membrane was incubated overnight at 4 °C with the following antibodies: PTEN, KLF4, AKT, p-AK, ERK1/2, pERK1/2 and GAPDH, followed by incubation with secondary antibodies. Immunoreactive bands were observed using an ultrasensitive chemiluminescence kit.

### Quantitative reverse transcription polymerase chain reaction (qRTPCR)

Total RNA was extracted from cells and exosomes using Trizol. miRNA reverse transcription was performed using the Mir-X miRNA First-Strand Synthesis Kit (Takara, Japan) and mRNA reverse transcription was performed using the PrimeScript™ RT reagent Kit (Takara, Japan) according to the instructions. reverse transcription using PrimeScript™ RT reagent Kit (Takara, Japan). Real-time PCR was performed using the Mx3000 Real-Time PCR Detection System (Agent, USA). 2^−ΔΔCt^ method was used to calculate the relative expression.

### Statistical analysis

All data are provided as mean ± SD. To compare data between two groups, the *t*-test for independent samples was employed, and one-way analysis of variance (ANOVA) was used to examine data between three or more groups. Statistical significance was defined as *p* < 0.05. Prism 9 software (GraphPad, USA) was used for statistical analysis.

### Ethics statement

All methods were carried out in accordance with relevant guidelines and regulations, and approved by the Ethics Committee of the First Hospital of Lanzhou University (LDYYLL-2024-01).

## Results

### Identification and characterization of hucMSCs and HUVEC

**Figure 1:**
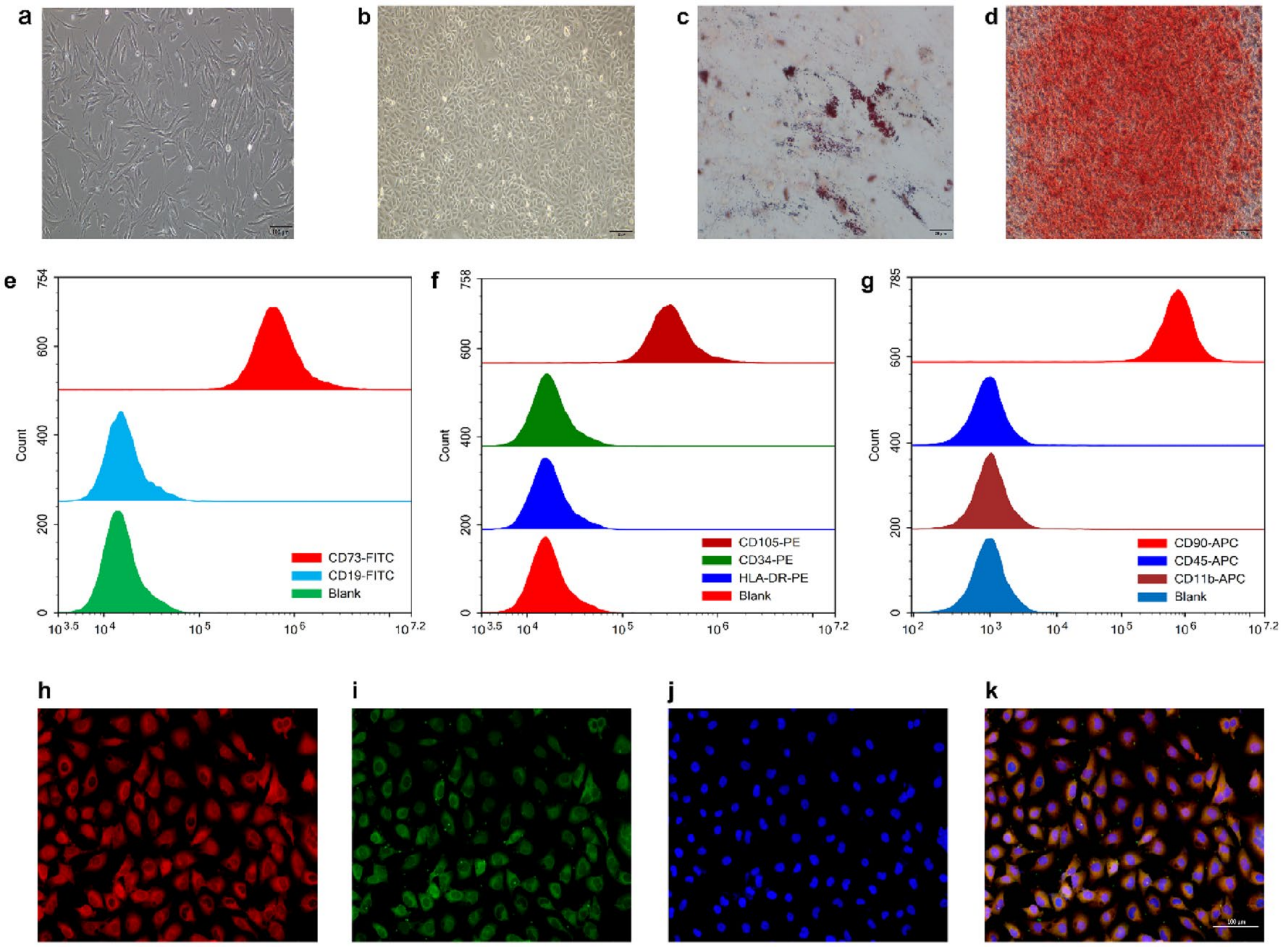
Identification and characterization of HUVECs and hucMSCs. (**a**) Representative pictures of hucMSCs. (**b**) Oil red O staining of hucMSCs for lipogenic differentiation ability. (**c**) Alizarin red staining of hucMSCs for osteogenic differentiation ability. (**d**) Representative pictures of HUVECs. (**e**) Flow cytometric results of CD73 and CD19, surface markers of hucMSCs. (**f**) Flow cytometric results for the surface markers CD34, CD105, and HLA-DR in hucMSCs. (**g**) Flow cytometry results of hucMSC surface markers CD11b, CD45, and CD90. (**h**) Immunofluorescence demonstration of HUVEC surface marker CD31 (red). (**i**) Immunofluorescence demonstration of HUVEC surface marker vWF (green). (**j**) DAPI staining (blue) of HUVEC nuclei. (**k**) Merged image of immunofluorescence staining of HUVECs.

Figure 1a,b show representative images of hucMSCs and HUVEC, respectively. Notably, hucMSCs showed fibroblast-like growth, and HUVECs showed epithelial cell-like growth. To identify the multispectral differentiation ability of hucMSCs, the differentiation of generated adipocytes and osteoblasts was identified by oil red O and alizarin red staining (Fig. 1c,d). Characterization of the surface markers of hucMSCs showed positive expression of CD73, CD90, and CD105 and negative expressions of CD11b, CD19, CD34, CD45, and HLA-DR (Fig. 1e–g). The immunofluorescence results showed that all HUVECs expressed CD31 and vWF (Fig. 1h–k). The experimental results demonstrated that both hucMSCs and HUVECs met the requirements of this experiment.

### Transfection and validation of miR-25-3p lentiviral vector

**Figure 2:**
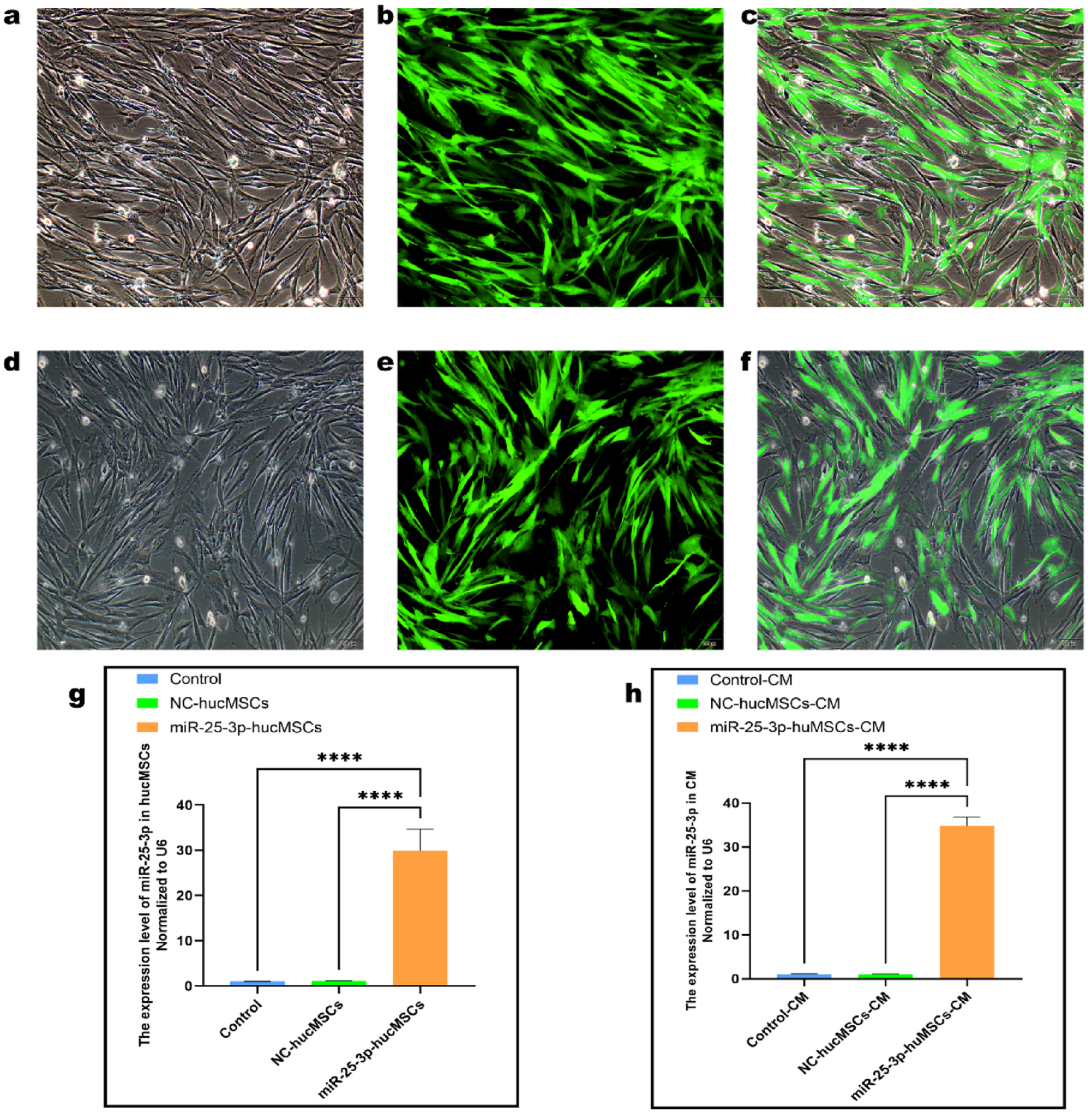
Transfection and validation of miR-25-3p lentiviral vector. (**a**) White light plot of the miR-25-3p-hucMSC group. (**b**) Green fluorescence plot of the miR-25-3p-hucMSC group. (**c**) Merged plot of the miR-25-3p-hucMSC group. (**d**) White fluorescence plot of the miR-25-3p-NC-hucMSC group. (**e**) Green fluorescence plot of the miR-25-3p-NC-hucMSC group. (**f**) Merge plot of the miR-25-3p-NC-hucMSC group. (**g**) RT-qPCR results of miR-25-3p transfection efficiency. (**h**) RT-qPCR expression results of miR-25-3p in the CM of cells in the control, miR-25-3p-NC-hucMSC, and miR-25-3p-hucMSC groups.

We transfected hucMSCs with miR-25-3p and miR-25-3p-NC lentiviral vectors, and the multiplicity of infection (MOI) values were set to 0, 20, 50, 80, or 100. When the MOI value was adjusted to 80, nearly all of the cells were green fluorescent protein (GFP)-positive (Fig. 2a–f). Therefore, a MOI of 80 was selected as the optimal transfection value. Subsequently, a reverse transcription-quantitative polymerase chain reaction (RT-qPCR) was performed to validate transfection efficiency. Notably, the expression of miR-25-3p in the miR-25-3p-hucMSCs group was 28-fold (*p* < 0.001; Fig. 2g) higher than that in the miR-25-3p-NC-hucMSCs and hucMSCs groups. In contrast, the expression of miR-25-3p in the cell supernatants was 33-fold (*p* < 0.001; Fig. 2h) higher in the miR-25-3p-hucMSCs group.

### Co-culture of miR-25-3p-modified hucMSCs and HUVECs

To investigate whether hucMSCs overexpression of miR-25-3p affects the function of HUVECs, cell co-cultures were used to probe the effects of miR-25-3p on the proliferation, migration and tubule-forming ability of HUVECs.

**Figure 3:**
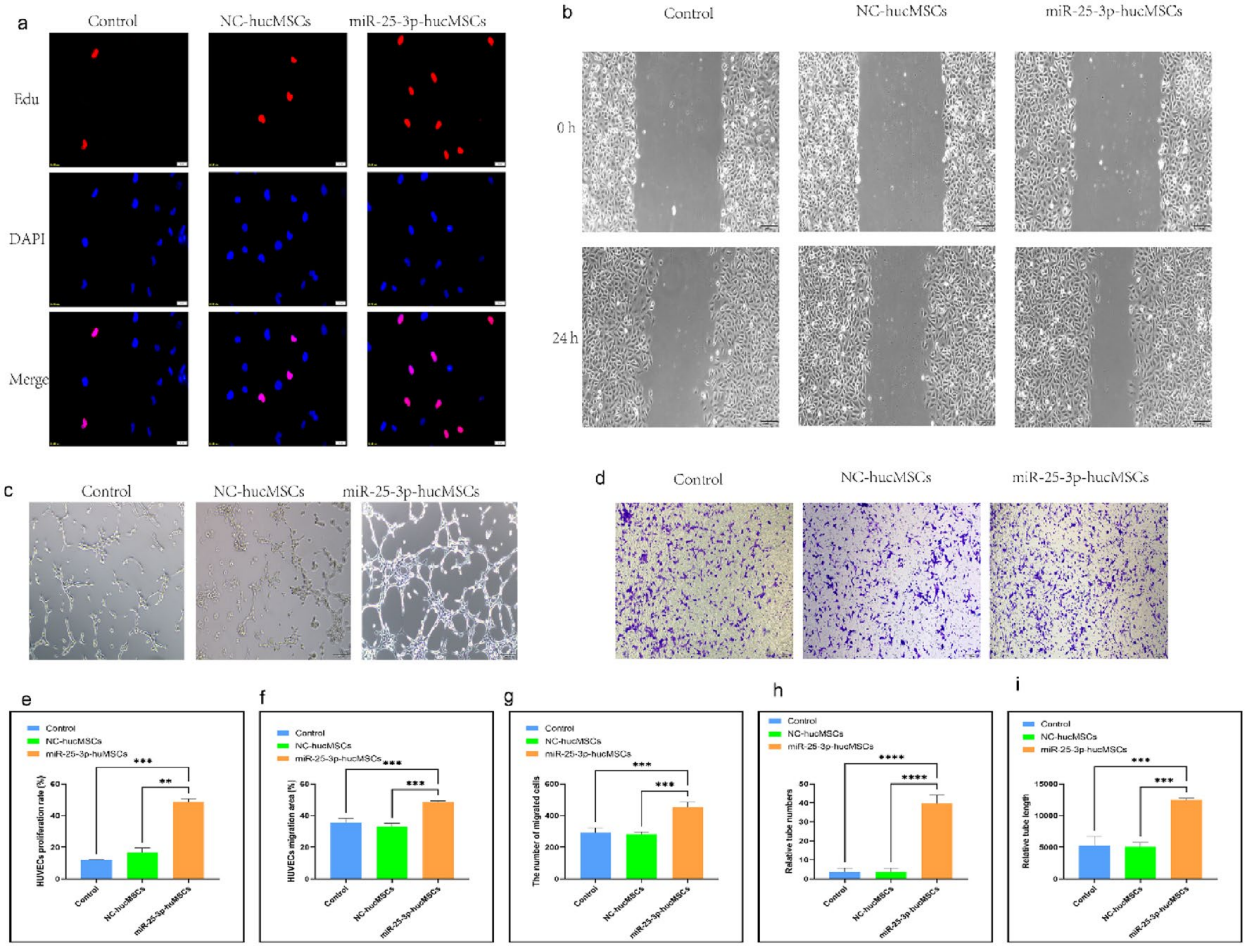
Co-culture with miRNA-25-3p-modified hucMSCs promotes HUVEC proliferation, migration, and tubule formation. (**a**) Representative images of the EdU proliferation experiment of HUVECs after co-culture. (**b**) Images of wound healing experiment of HUVECs after co-culture. (**c**) Images of tubule formation experiment of HUVECs after co-culture. (**d**) Images of transwell experiment of HUVECs after co-culture. (**e**) Quantification of EdU proliferation experiment of HUVECs. (**f**) Quantification of the area of the migrated region of HUVECs. (**g**) Quantification of the number of migrated HUVEC cells using the transwell assay. Quantification of the number of tubules (**h**) and tubule length (**i**) in HUVECs using a tube formation assay.

First, the proliferative ability of HUVECs after co-culture was examined using an Edu proliferation kit. The EdU proliferation assay findings demonstrated that the positive rate of proliferating HUVECs in the miR-25-3p-hucMSC group was significantly higher than that in the control and miR-25-3p-NC-hucMSC groups, whereas that in the control and miR-25-3p-NC-hucMSCs groups was not statistically different (*p* < 0.001, Fig. 3a,e).

Second, scratch and transwell experiments were performed to verify the migratory capacity of HUVECs after co-culture. The scratch assay showed that miR-25-3p-modified hucMSCs significantly increased the migratory area of HUVECs (Fig. 3b,f). Similarly, the transwell assay showed that the number of HUVECs on the bottom side of the transwell chambers significantly increased in the miR-25-3p-hucMSC group (Fig. 3d,g).

Finally, tubule formation experiments were performed to verify the angiogenic capacity of HUVECs after co-culture. Notably, the number and length of formed tubules in HUVECs were significantly increased in the miR-25-3p-NC-hucMSC group compared with those in the control and miR-25-3p-NC-hucMSC groups (Fig. 3c,h,i). Therefore, miRNA126-3p-modified hucMSCs enhanced the proliferation, migration, and angiogenesis of co-cultured HUVECs through the paracrine pathway.

### Conditioned medium (CM) from miR-25-3p-modified hucMSCs used for intervention in HUVECs

We treated HUVECs with CM to confirm whether miR-25-3p-modified hucMSCs affected the proliferation, migration, and tubule-forming abilities of HUVECs through the paracrine pathway.

**Figure 4:**
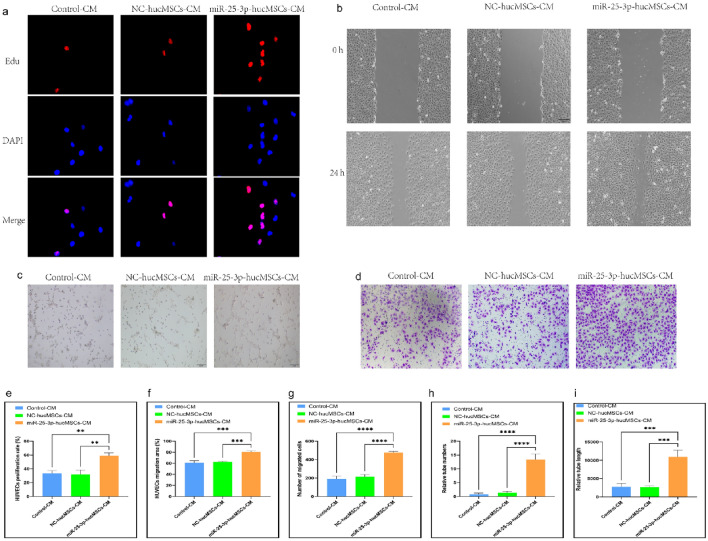
miRNA-25-3p-modified hucMSCs-derived CM promotes HUVEC proliferation, migration, and tubule formation. (**a**) Representative images of the EdU proliferation assay of HUVECs. (**b**) Images of wound healing assay of HUVECs. (**c**) Images of tubule formation assay of HUVECs. (**d**) Images of transwell assay of HUVECs. (**e**) Quantification of EdU proliferation experiment of HUVECs. (**f**) Quantification of the area of the migrated region of HUVECs. (**g**) Quantification of the number of migrated HUVEC cells using the transwell assay. Quantification of the number of tubules (**h**) and tubule length (**i**) in HUVECs using a tube formation assay.

The EdU proliferation assay was used to examine the proliferative capacity of HUVECs after CM intervention. Notably, the positive rate of proliferating HUVECs significantly increased with the CM intervention in the miR-25-3p-hucMSC group than in the control and miR-25-3p-NC-hucMSC groups (Fig. 4a,e).

Subsequently, scratch and transwell assays were performed to verify the migration ability of HUVECs after CM intervention. The scratch assay results showed that miR-25-3p-modified hucMSC-derived CM significantly increased the migratory area of HUVECs (Fig. 4b,f). Similarly, the results of the transwell assay showed that CM in the miR-25-3p-hucMSCs group significantly increased the number of HUVECs on the bottom surface of the transwell chamber (Fig. 4d,g).

Finally, the angiogenic capacity of HUVECs after CM intervention was verified using a tubule formation assay. Notably, the CM in the miR-25-3p-NC-hucMSC group significantly increased the number and length of tubules formed by HUVECs compared with the CM in the control and miR-25-3p-NC-hucMSC groups (Fig. 4c,h,i). Therefore, miRNA126-3p-modified mucMSCs could promote the proliferation, migration, and angiogenesis of co-cultured HUVECs through the paracrine pathway.

### Effect of miRNA-25-3p-modified hucMSC-derived exos intervention on HUVEC

We observed that hucMSCs influence the function of HUVEC through a paracrine pathway. Exos can affect the functions of other cells by transporting miRNAs. We intervened in HUVECs with exosomes from CM to investigate whether miR-25-3p affects the proliferation, migration, and angiogenic capacity of HUVEC through the exosomal pathway.

**Figure 5:**
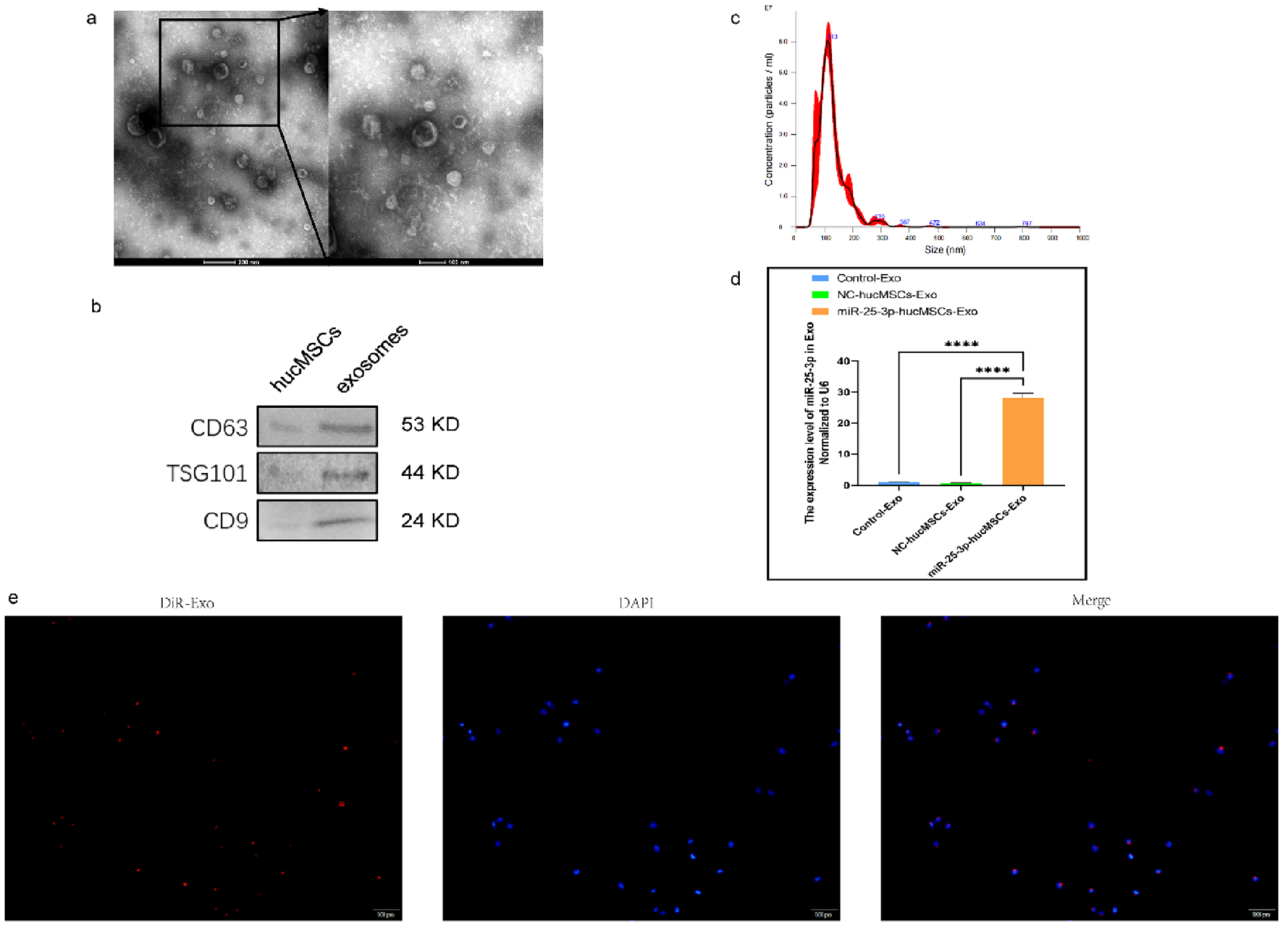
Exosome characterization and identification. (**a**) Morphology of exosomes. (**b**) Western blot results of markers on the surface of exosomes. (**c**) Results of NTA analysis of exosomes. (**d**) Expression of miRNA-25-3p in exosomes. (**e**) DiR-labeled exosomes derived from hucMSCs (red) can be internalized by DAPI-labeled HUVECs (blue).

First, we extracted the exos and characterized their morphology using transmission electron microscopy (Fig. 5a) and the surface markers CD9, CD63, and TSG101 using western blotting (Fig. 5b). Nanoparticle tracking analysis (NTA) was used to determine the diameter of the exos (Fig. 5c). RT-qPCR was used to verify the differential expression of miR-25-3p in exos. Notably, miR-25-3p expression was 27-fold greater in exos produced from miR-25-3p-hucMSCs than in exos obtained from the other two groups. (Fig. 5d). Subsequently, in vitro tracking assays were performed to determine whether HUVECs could take up Dil-labeled exos. Notably, the exos were successfully internalized by HUVECs (Fig. 5e).

**Figure 6:**
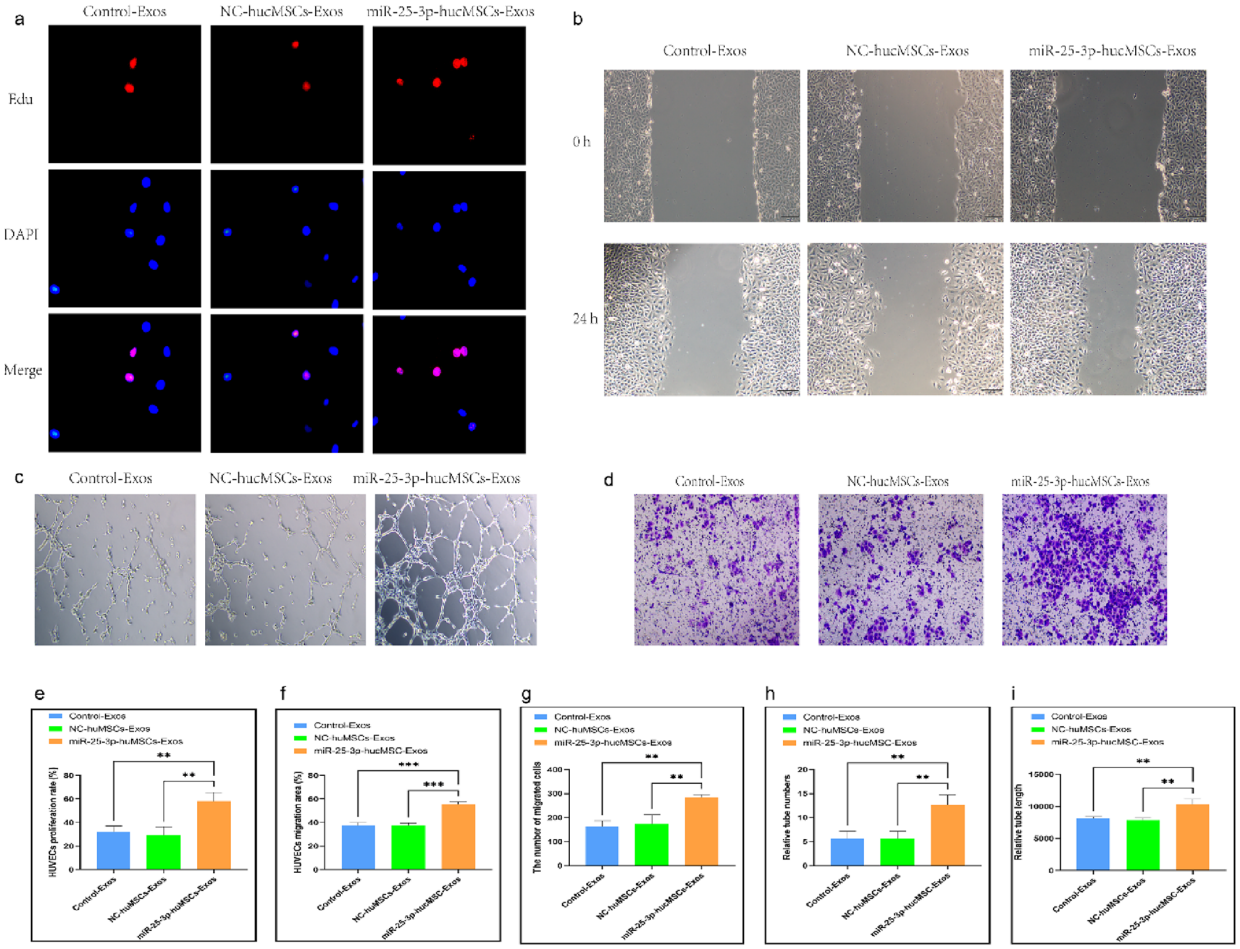
miRNA-25-3p modified hucMSCs-derived exosomes promote HUVEC proliferation, migration, and tubule formation. (**a**) Representative images of the EdU proliferation assay of HUVECs. (**b**) Images of wound healing assay of HUVECs. (**c**) Images of tubule formation assay of HUVECs. (**d**) Images of transwell assay of HUVECs. (**e**) Quantification of EdU proliferation experiment of HUVECs. (**f**) Quantification of the area of the migrated region of HUVECs. (**g**) Quantification of the number of migrated HUVEC cells using the transwell assay. Quantification of the number of tubules (**h**) and tubule length (**i**) in HUVECs using a tube formation assay.

To investigate the effects of exos derived from miR-25-3p-modified hucMSCs on the proliferation, migration, and tubule-forming ability of HUVECs, HUVECs were given 50 μg/ml of exos derived from the control, miR-25-3p-NC-hucMSC, and miRNA-25-3p-hucMSC groups. Compared with the control and miR-25-3p-NC-hucMSC groups, exos derived from the miR-25-3p-hucMSC group significantly increased proliferation (Fig. 6a,e), migration capacity (Fig. 6b,f,d,g), and in vitro angiogenic capacity (Fig. 6c,h,i) of HUVECs. Therefore, miR-25-3p-modified hucMSCs can affect HUVEC function through the exosomal delivery of miR-25-3p.

### Neutral sphingomyelinase 2 inhibitor (GW4869) affects HUVEC function by decreasing secretions of exos

**Figure 7:**
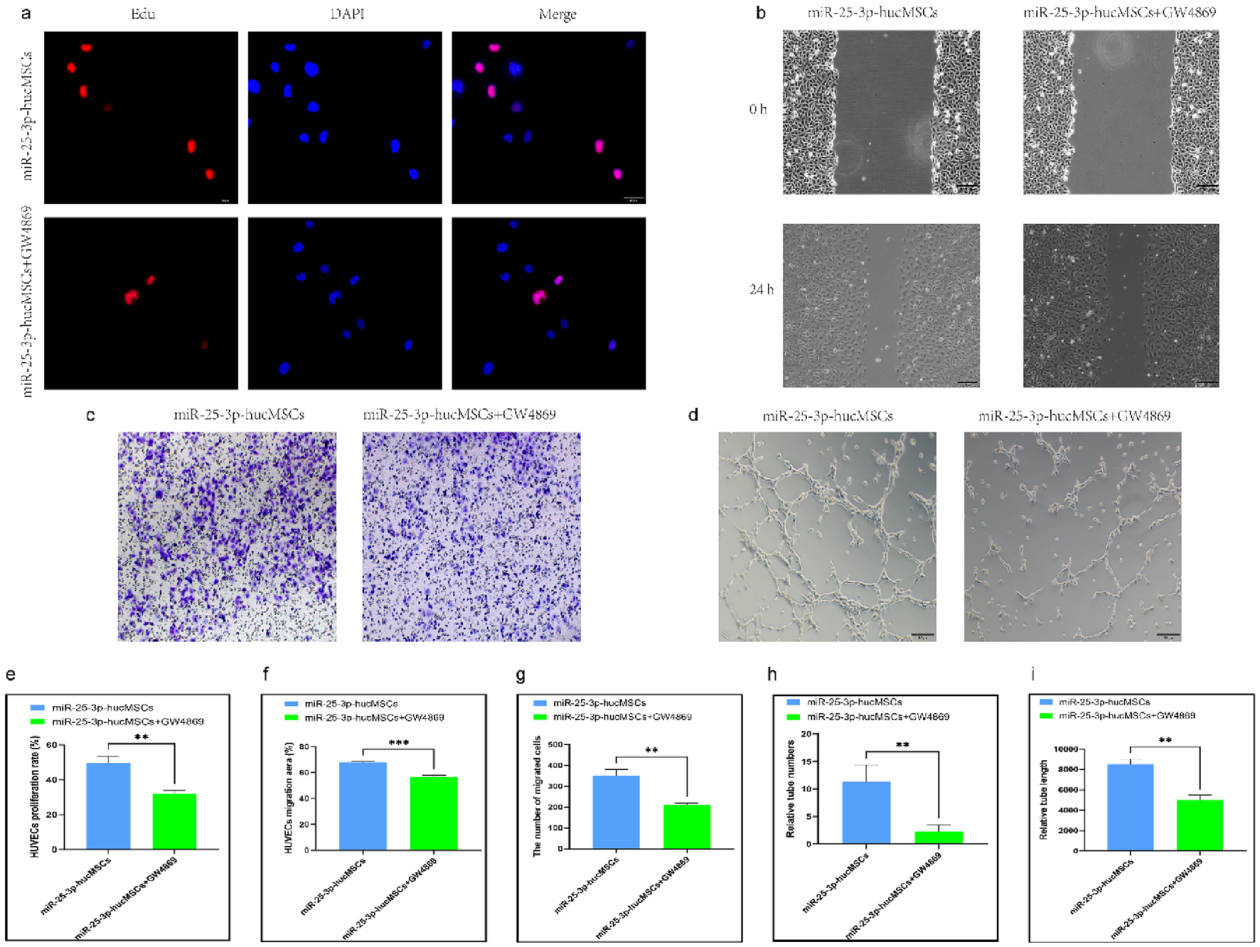
GW4869 inhibits HUVEC function after co-culture with miR-25-3p-hucMSCs. (**a**) Representative images of the EdU proliferation assay of HUVECs. (**b**) Images of wound healing assay of HUVECs. (**c**) Images of transwell assay of HUVECs. (**d**) Images of tubule formation assay of HUVECs. (**e**) Quantification of EdU proliferation experiment of HUVECs. (**f**) Quantification of the area of the migrated region of HUVECs. (**g**) Quantification of the number of migrated HUVEC cells using the transwell assay. Quantification of the number of tubules (**h**) and tubule length (**i**) in HUVECs using a tube formation assay.

To verify whether miR-25-3p-modified hucMSCs influenced HUVEC function through exosomal secretion, we investigated the effect of exosome release inhibitors. The co-cultured HUVECs in the miR-25-3p-hucMSCs + GW4869 group showed significantly decreased proliferation (Fig. 7a,e) and migration (Fig. 7b,c,f,g) and reduced in vitro angiogenic capacity compared with the miR-25-3p-hucMSC group (Fig. 7d,h,i).

**Figure 8:**
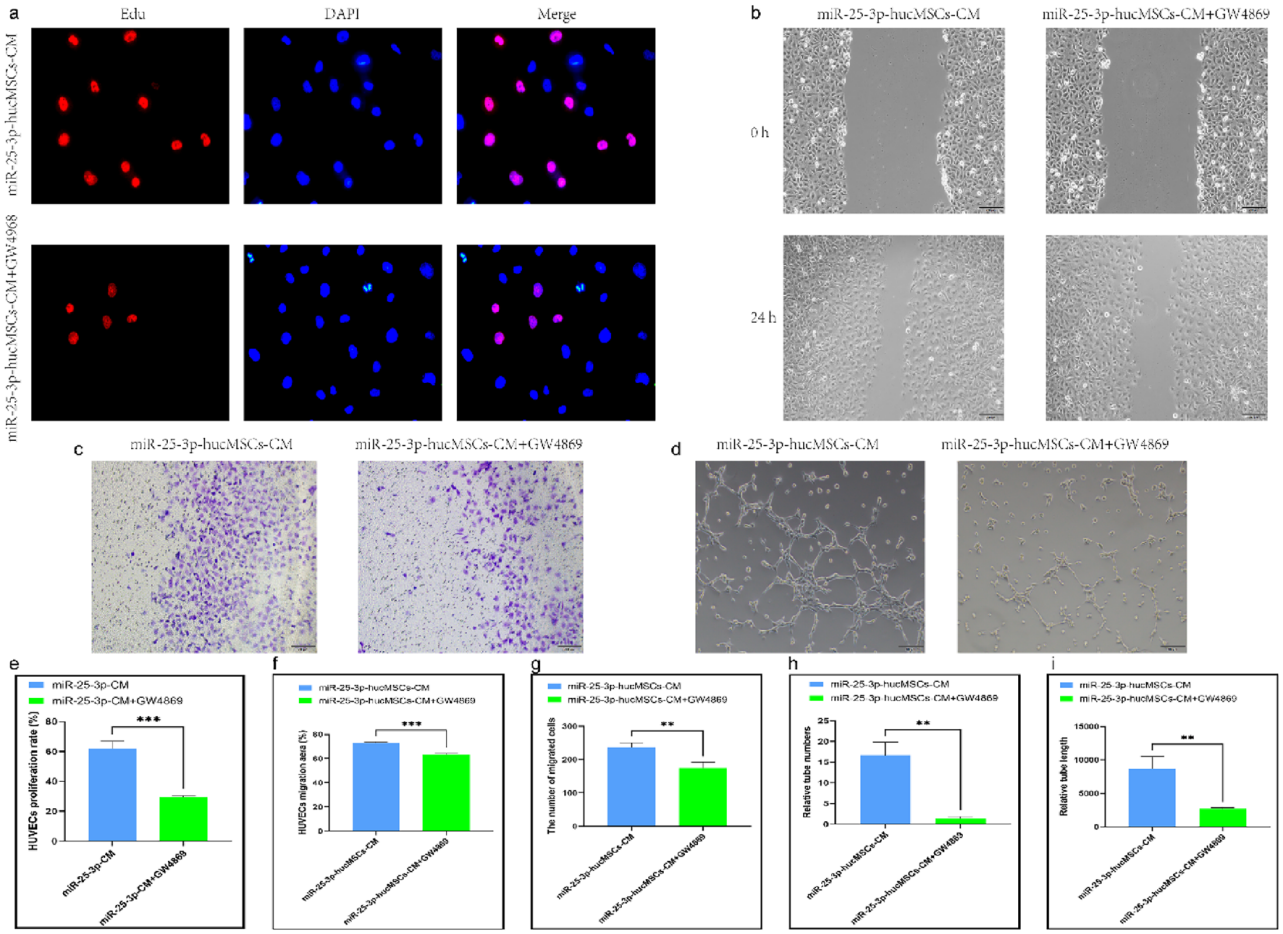
GW4869 inhibits function of HUVECs after intervening with miR-25-3p-hucMSCs-derived CM. (**a**) Representative images of the EdU proliferation assay of HUVECs. (**b**) Images of wound healing assay of HUVECs. (**c**) Images of Transwell assay of HUVECs. (**d**) Images of tubule formation assay of HUVECs. (**e**) Quantification of EdU proliferation experiment of HUVECs. (**f**) Quantification of the area of the migrated region of HUVECs. (**g**) Quantification of the number of migrated HUVEC cells using the transwell assay. Quantification of the number of tubules (**h**) and tubule length (**i**) in HUVECs using a tube formation assay.

Similarly, HUVECs treated with CM in the miR-25-3p-hucMSCs + GW4869 group displayed considerably decreased proliferative activity (Fig. 8a,e) and migratory (Fig. 8b,c,f,g) and tubule-forming capacities than those in the miR-25-3p-hucMSCs group (Fig. 8d,h,i).

### Exos derived from miR-25-3p-modified hucMSCs regulate cellular function of HUVECs by targeting KLF4 and PTEN in HUVEC

**Figure 9:**
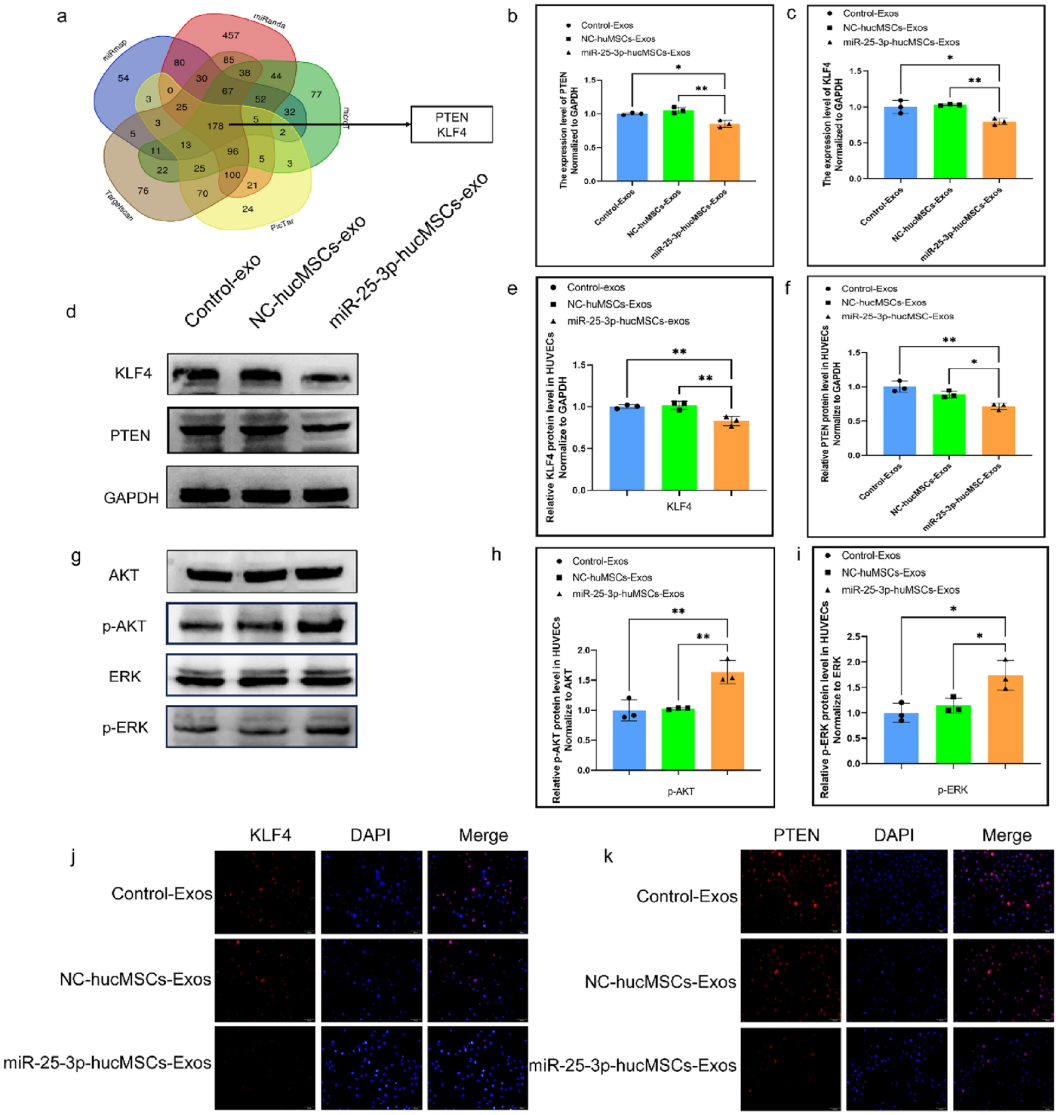
miR-25-3p-modified hucMSCs-derived exosomes regulate HUVECs function by targeting PTEN and KLF4. (**a**) Bioinformatic analysis of potential target genes of miR-25-3p. (**b**) The mRNA expression level of PTEN in HUVECs after exosomes intervention. (**c**) The mRNA expression level KLF4 in HUVECs after exosomes intervention. (**d**) The protein expression levels of PTEN and KLF4 in HUVECs after exosome intervention. (**e**,**f**) Quantification of PTEN and KLF4 protein expression levels. (**g**) The protein expression levels of AKT phosphorylation and ERK1/2 phosphorylation in HUVECs after exosome intervention. (**h**,**i**) Quantification of AKT phosphorylation and ERK1/2 phosphorylation protein expression levels. (**j**,**k**) Cytofluorescence verification of PTEN and KLF4 protein expression levels in HUVEC.

Following bioinformatics analysis and synthesis of prior studies^23,24^, we selected KLF4 and PTEN as probable downstream target genes of miRNA-25-3p for further research. (Fig. 9a). We extracted total proteins from HUVECs after exosome treatment for subsequent studies. The mRNA and protein expression levels of KLF4 and PTEN were significantly lower in the miRNA-25-3p-MSC group compared with the hucMSC and miRNA-25-3p-NC-hucMSC groups (Fig. 9b–f). Furthermore, the miRNA-25-3p-hucMSC group had considerably higher levels of phosphorylated AKT and ERK1/2. (Fig. 9g–i). Finally, immunofluorescence showed that PTEN and KLF4 protein levels were significantly reduced in the miR-25-3p-hucMSC-Exos group (Fig. 9j,k).

**Figure 10:**
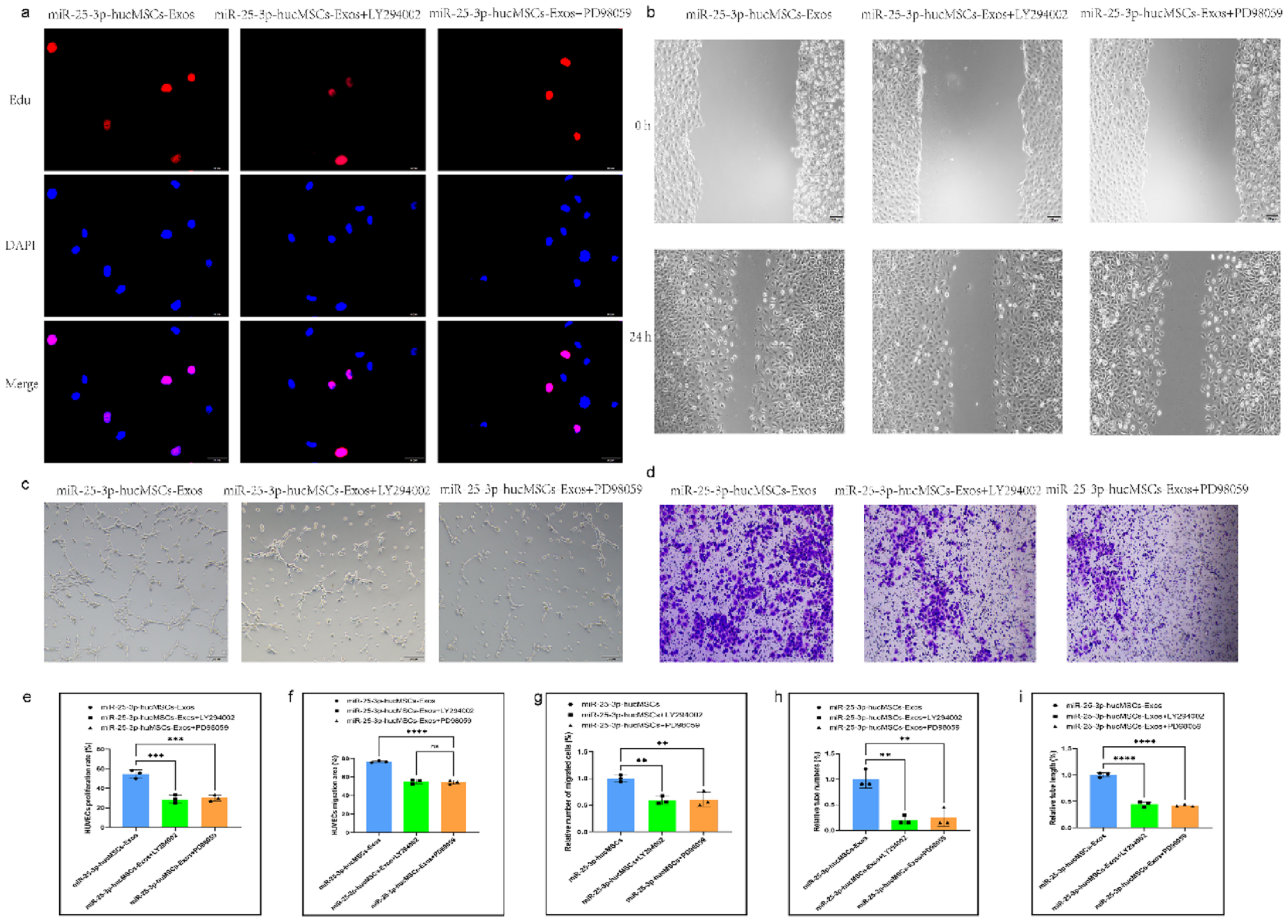
Signaling pathway inhibitors reversed HUVEC function in exosomal intervention experiments. (**a**) Representative images of the EdU proliferation assay of HUVECs. (**b**) Images of wound healing assay of HUVECs. (**c**) Images of tubule formation assay of HUVECs. (**d**) Images of transwell assay of HUVECs. (**e**) Quantification of EdU proliferation experiment of HUVECs. (**f**) Quantification of the area of the migrated region of HUVECs. (**g**) Quantification of the number of migrated HUVEC cells using the transwell assay. Quantification of the number of tubules (**h**) and tubule length (**i**) in HUVECs using a tube formation assay.

In addition, to validate the aforementioned pathways, we used the AKT inhibitor LY294002 and the ERK1/2 inhibitor PD098059 in the exosomal intervention studies. The AKT inhibitor LY294002 and the ERK1/2 inhibitor PD098059, in particular, dramatically decreased proliferation (Fig. 10a,e) and migration ability (Fig. 10b,c,f,g) of HUVECs and also reduced the number and length of tubes formed in HUVECs compared with the miR-25-3p-hucMSC group (Fig. 10d,h,i).

### Transplantation of miR-25-3p-modified hucMSCs in the PVT model

All animal procedures were undertaken in accordance with relevant guidelines and regulations, were approved by the Ethics Committee of the First Hospital of Lanzhou University. Findings and experiments described in this paper were designed and reported following the Animal Research: Reporting of In Vivo Experiments (ARRIVE) guidelines.

**Figure 11:**
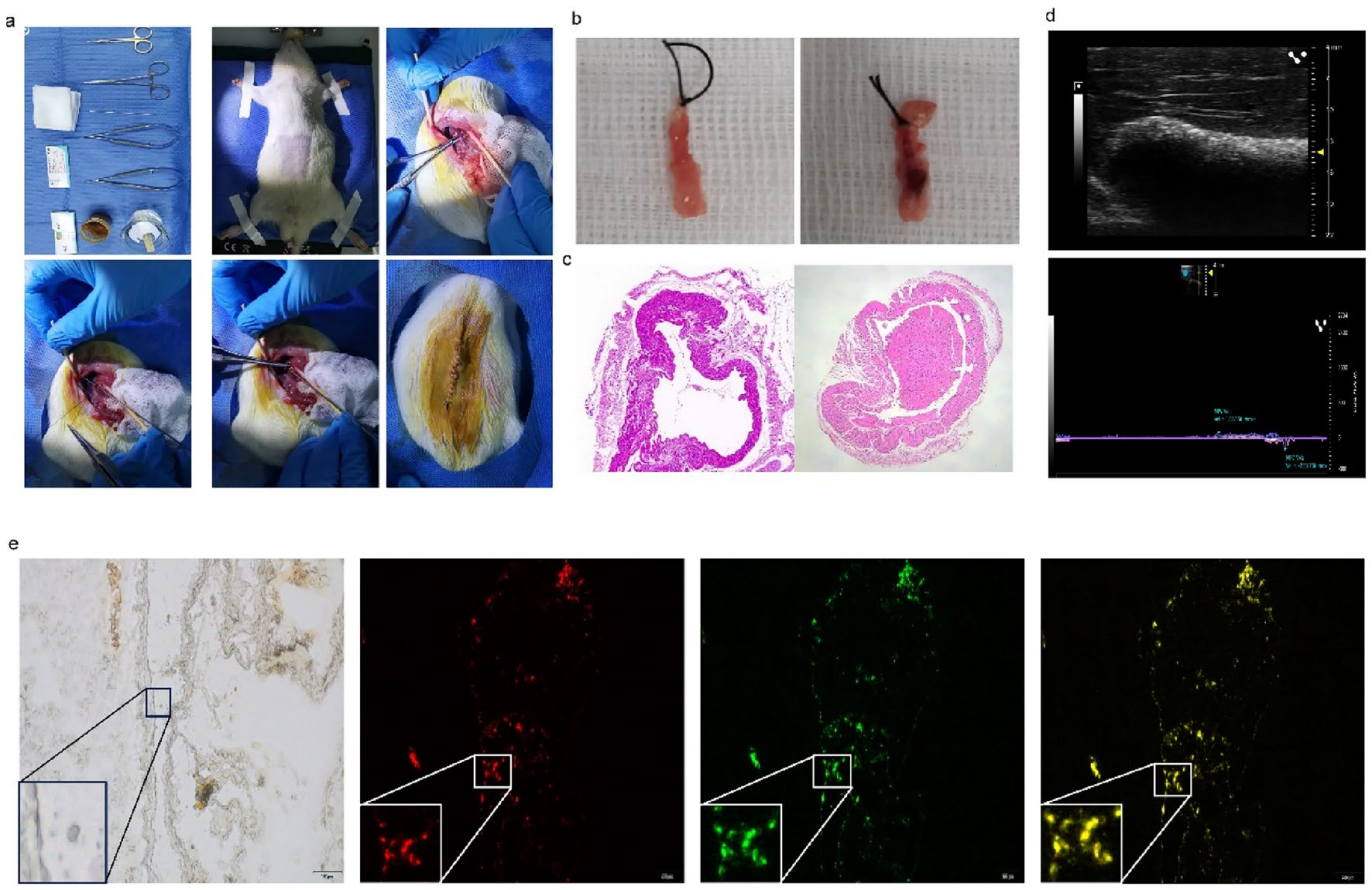
Transplantation of miR-25-3p-modified hucMSCs in vivo. (**a**) Construction of the rat PVT model. (**b**) Representative picture of the portal vein under the stereomicroscope. (**c**) Representative pictures of the pathology of portal vein thrombosis. (**d**) Representative images of portal vein ultrasound. (**e**) Representative images of miR-25-3p-modified hucMSCs homing.

To determine whether miRNA-25-3p overexpression in hucMSCs improved portal endothelial cell repair and reduced the local inflammatory response in vivo, we used a rat PVT model for further validation. First, we constructed (Fig. 11a) and validated a rat PVT model (Fig. 11b,c). Detection of portal vein flow rate and internal diameter by small animal ultrasonography (Fig. 11d). The homing of the implanted hucMSCs was tracked via Dil labeling. The homing of the implanted hucMSCs was tracked via Dil labeling. Immunofluorescence studies showed that Dil-positive cells were predominantly located in the damaged portal vein endothelium (Fig. 11e). The damaged portal veins in the miRNA-25-3p-hucMSCs group had a higher degree of repair than those in the other groups.

### In vivo transplantation of miRNA-25-3p-modified hucMSC promotes the repair of damaged portal veins

**Figure 12:**
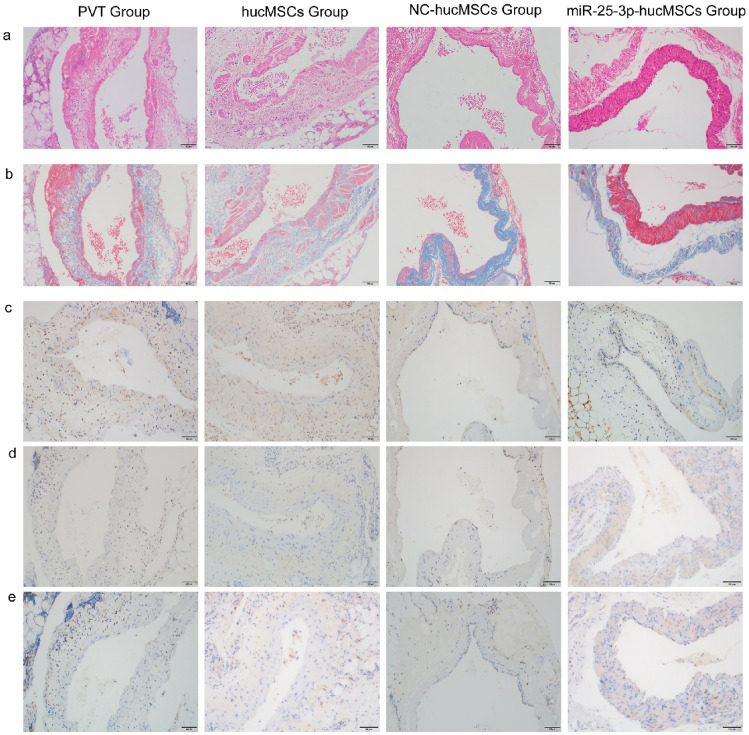
In vivo transplantation of miRNA-25-3p-modified hucMSCs attenuated rat portal vein inflammation and promoted endothelial cell proliferation. (**a**) Representative image of the portal vein stained with H&E. (**b**) Representative image of the portal vein stained with Masson trichrome. (**c**) Representative image of the portal vein stained with TNF-α. (**d**) Representative image of the portal vein stained for Ki67. (**e**) Representative image of the portal vein stained for CD68.

To further validate the role of miRNA-25-3p-modified hucMSCs, we transplanted miRNA-25-3p-modified hucMSCs in vivo and observed the repair of portal veins. We obtained portal veins from each group and performed hematoxylin and eosin (H&E; Fig. 12a) and Masson (Fig. 12b) staining to observe the repair of portal veins. Additionally, we performed immunohistochemical staining for Ki-67 (Fig. 12c) in the portal veins of each group to observe the proliferation of venous endothelial cells. Simultaneously, staining for TNF-α (Fig. 12d) and CD68 (Fig. 12e) was performed to observe the inflammatory factors in the portal veins and infiltration of inflammatory cells, respectively. In vivo transplantation of miRNA-25-3p-hucMSC enhanced endothelial cell proliferation while decreasing TNF-α and CD68-positive cell expression in portal veins.

Therefore, in vivo transplantation of miRNA-25-3p-hucMSC may have a favorable protective effect on damaged portal veins compared with treatment with hucMSCs or miR-25-3p-NC-hucMSCs.

## Discussion

Cirrhotic patients with PVT have been reported to have a 55% lower 2-year survival rate due to abnormal liver function^25^. Therefore, the delicate balance between addressing bleeding disorders and administering anticoagulation is crucial in the treatment of PVT in patients with cirrhosis. As endothelial cell injury plays a pivotal role in thrombosis^26^, the repair of damaged venous endothelial cells and promotion of endothelial patency, which in turn facilitates thrombus recanalization, are essential.

In vitro cellular function experiments revealed that miRNA-25-3p-modified hucMSCs secreted exos to enhance endothelial cell proliferation, migration, and tubule formation. In addition, we explored the potential target genes and signaling pathways and validated that miR-25-3p-modified hucMSCs promoted the proliferation of venous endothelial cells, attenuated the infiltration of inflammatory factors and cells, and promoted the repair of endothelial cell injury in vivo using a PVT rat model.

Stem cells are currently attracting attention in the field of regenerative medicine and the treatment of vascular diseases owing to their autopoietic properties^27–29^. In particular, MSC-derived exos may exhibit better anti-inflammatory and tissue-repairing effects owing to their small size, better biocompatibility, and lower immunogenicity^30^. In addition, exos may contain extracellular matrix proteins, metabolites, mRNA, noncoding RNA, and DNA and play an important role in intercellular communication^31–33^. Targeted peptide-modified hucMSC-derived exos have shown improved targeting of hepatic stellate cells to attenuate liver fibrosis^20^. Similarly, surface-modified engineered exos better attenuate cerebrovascular ischemia–reperfusion injury by targeting quercetin delivery to damaged neurons^34^. Therefore, exos may serve as good transport carriers for tissue repair and disease treatment^35^.

In addition, miRNAs carried by MSC exos play an important role in the treatment of vascular diseases^36^. For example, miRNA-126-3p-modified exos derived from hucMSCs promote re-endothelialization of grafted veins^37^. Further, hucMSC-derived exos harboring miR-342-3p inhibit deep vein thrombosis by down-regulating EDNRA^22^ and protect the vascular endothelium from diabetic damage by affecting MAPK/ERK signaling^38^.

In addition, there are other questions that need to be explored in the article's experiments. There may be more target genes and pathways that miRNA-25-3p can potentially act on, and we may subsequently validate and explore other target genes and corresponding pathways. We explored that miR-25-3p-modified exosomes derived from human umbilical cord mesenchymal stem cells could promote the proliferation, migration, and angiogenic capacity of human umbilical vein endothelial cells through the PTEN/KLF4/AKT/ERK1/2 pathway. The in vivo mechanism of miR-25-3p overexpressed hucMSCs mediating vascular endothelial cell repair through an exosomal mechanism will be further explored in the subsequent studies of our group.

## Conclusion

In conclusion, this study revealed that miRNA-25-3p overexpression in hucMSCs enhanced endothelial function through an exosomal mechanism. In vivo transplantation of cells attenuates inflammatory factors and cellular infiltration while promoting proliferation and repair of venous endothelial cells. Thus, in vivo infusion of genetically modified stem cell-derived exosomes is expected to be a potential therapy for PVT treatment.

### Supplementary Information

Supplementary Information.

## Data Availability

Any additional information required to reanalyze the data reported in this paper is available from the corresponding author upon request.
