# Supplementary material for: Enhancement of endothelial function and attenuation of portal vein injury using mesenchymal stem cells carrying miRNA-25-3p
Source: Sci Rep. 2024 Jul 2;14:15113. doi: 10.1038/s41598-024-64263-6 (PMC11220092; doi:10.1038/s41598-024-64263-6)

Supplementary raw data of western blotting

Figure-5

1): CD63:

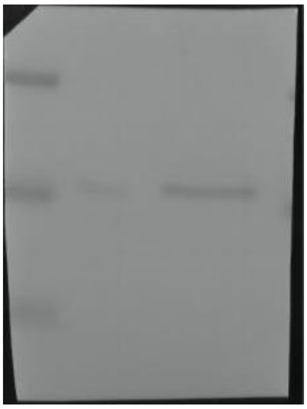

2): CD9:

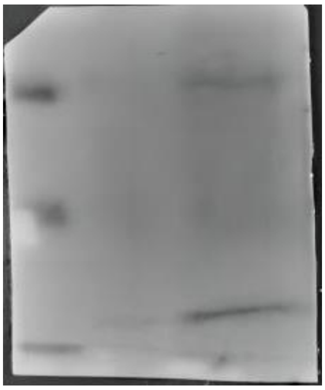

3): TSG101:

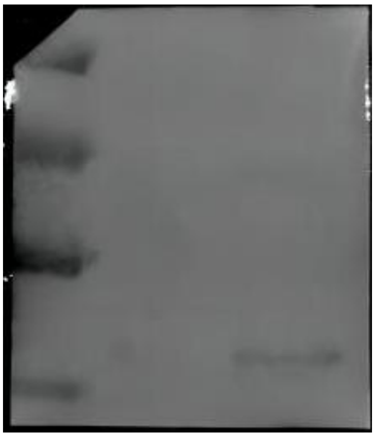

Figure-9

1) PTEN:

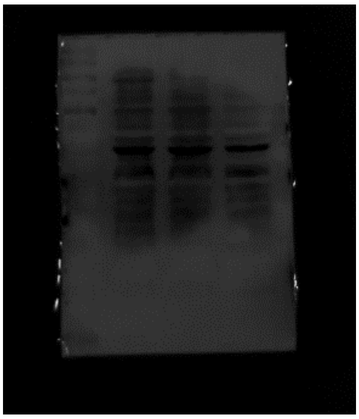

2) KLF4:

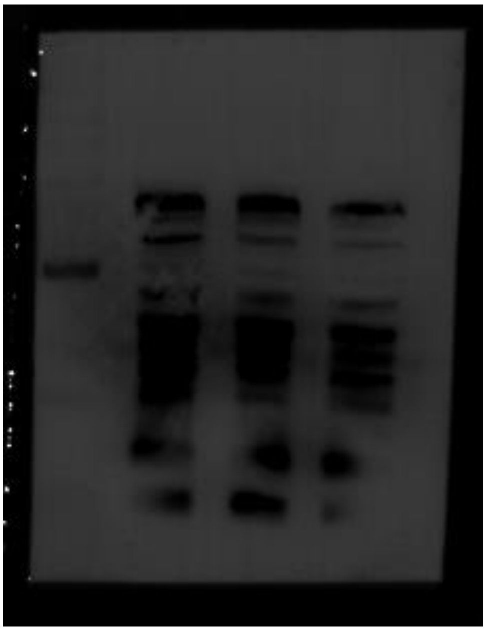

3) GAPDH

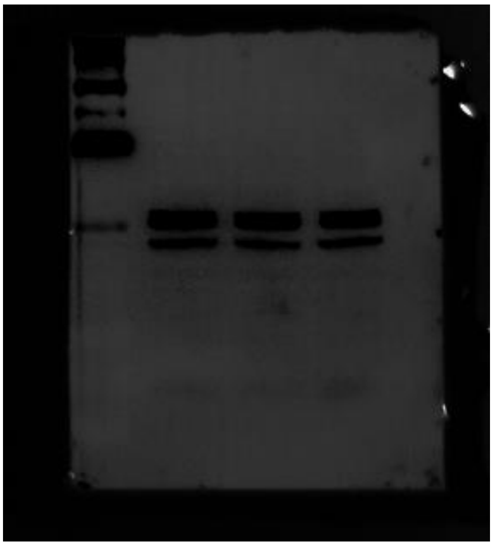

4) AKT

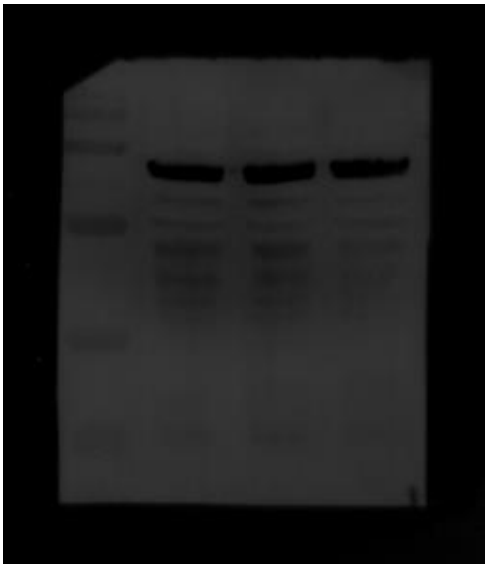

5) p-AKT

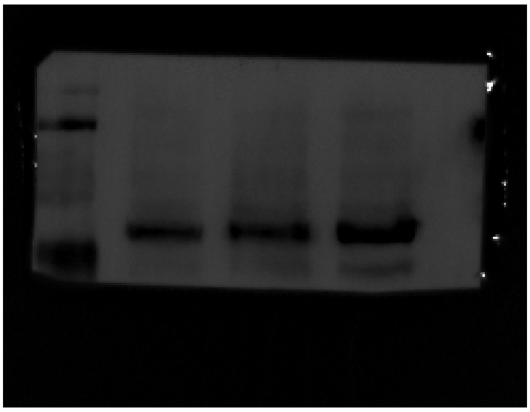

6) ERK

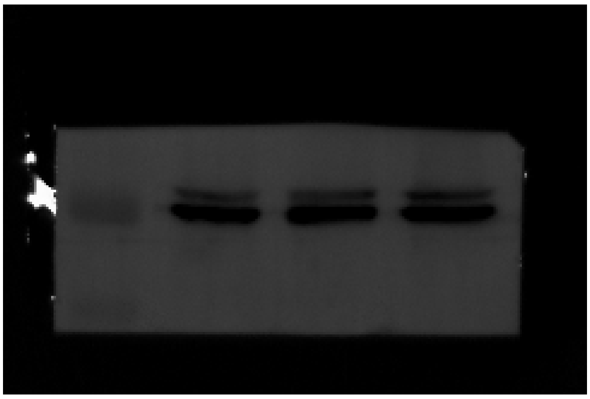

7) p-ERK

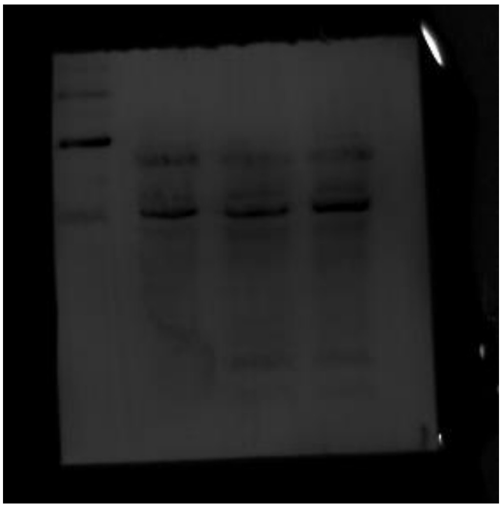

Supplement: Supplementary file 1 — Supplementary Information. [file 41598_2024_64263_MOESM1_ESM.pdf]
